# A Systematic Review on Recent Advances in mHealth Systems: Deployment Architecture for Emergency Response

**DOI:** 10.1155/2017/9186270

**Published:** 2017-09-17

**Authors:** Enrique Gonzalez, Raul Peña, Alfonso Avila, Cesar Vargas-Rosales, David Munoz-Rodriguez

**Affiliations:** Tecnologico de Monterrey, 64849 Monterrey, NL, Mexico

## Abstract

The continuous technological advances in favor of mHealth represent a key factor in the improvement of medical emergency services. This systematic review presents the identification, study, and classification of the most up-to-date approaches surrounding the deployment of architectures for mHealth. Our review includes 25 articles obtained from databases such as IEEE Xplore, Scopus, SpringerLink, ScienceDirect, and SAGE. This review focused on studies addressing mHealth systems for outdoor emergency situations. In 60% of the articles, the deployment architecture relied in the connective infrastructure associated with emergent technologies such as cloud services, distributed services, Internet-of-things, machine-to-machine, vehicular ad hoc network, and service-oriented architecture. In 40% of the literature review, the deployment architecture for mHealth considered traditional connective infrastructure. Only 20% of the studies implemented an energy consumption protocol to extend system lifetime. We concluded that there is a need for more integrated solutions specifically for outdoor scenarios. Energy consumption protocols are needed to be implemented and evaluated. Emergent connective technologies are redefining the information management and overcome traditional technologies.

## 1. Introduction

Efforts and developments for pervasive healthcare are growing swiftly worldwide; this fact reveals that universities, researchers, and enterprises are aware of the need for functional and accurate systems that are able to satisfy the increasing demand for a better medical attention infrastructure [1]. Although healthcare monitoring systems are being gradually adopted, their performance in clinical settings is still controversial [2]. Despite this controversial performance, technology has changed the vision of how caregivers should react under medical emergencies. Traffic accidents have become a major cause of death around the world, demanding better emergency care services. In 2012, the World Health Organization (WHO) estimated that traffic accidents were the leading cause of death in people between the ages of 15 and 29. The WHO also predicted that traffic accidents would become the seventh leading cause of death around the world by 2030 [3]. Additionally, people suffering chronic and degenerative diseases, especially elderly people [4], also demand better healthcare services. Diabetes, high blood pressure, and cardiovascular disorders are examples of chronic and degenerative diseases that limit people from doing their daily outdoor activities. Therefore, the availability of improved emergency and healthcare services is the technological challenge that can let people to live in a safe and independent way.

Health monitoring is one of the ways to improve the emergency and healthcare services. Heath monitoring enables the early detection of diseases and the prompt medical care under emergencies resulting in the reduction of suffering and medical costs. The use of sensors for monitoring and transmitting the patient's vital signs is useful for identification of patients under risk. The medical personnel can determine the actions to safeguard the patient's health using the sensor information. The delivery of opportune medical care could be the difference between life and death.

Advances in mHealth services incorporate platforms based on wireless body sensor networks (WBSN) as a central aid for remote reporting of data to a medical center under medical emergencies. The WBSN collects data from biomedical sensors and cameras using a user-friendly interface. The WBSN is also able to transmit images, physiological signals, and video [5]. In [6], the authors present the Emergency Remote Pre-Hospital Assistance platform. This platform enables medical surveillance in real time from accident location.

### 1.1. Emergency Situations

An emergency condition can be caused by multiple and different factors, such as natural disasters, human carelessness, explosions, traffic accidents, and degenerative diseases. On these events, early attention is very important, also to have a plan of action, and, of course, the caregiver capabilities. Once the caregivers are notified about the emergency situation, the prehospital assistance starts with the intention to control the emergency by stabilizing the patient for further transportation to a medical center.

Events resulting in an emergency condition are natural disasters, human carelessness, explosion, traffic accidents, degenerative diseases, and so forth. These events may be may be multiple and different. Addressing these events also require (1) early attention, (2) a plan of action, and (3) a capable caregiver.

After notifying the caregiver about the emergency, the prehospital assistance starts with the intention of stabilizing the patient for further transportation to a medical center.

Providing first aid and requesting the assistance of a specialist physician at the same time increases the chances of a better outcome for patients in critical conditions. To do this, the assistance request to the specialist physician must include an accurate overview of the patient. This overview should be prepared by collecting and sending patient's information in real time.

**Figure 1:**
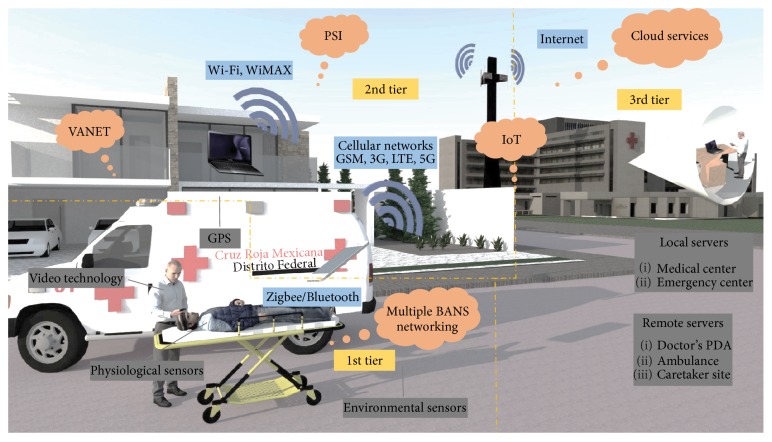
Conceptual mHealth system architecture for emergency situation in outdoors.

Under this emergency scenario, the incorporation of a wireless body sensor network (WBSN) enables real-time monitoring and results in delivering better and opportunistic medical services. Nevertheless, developing this integrated system for emergency care becomes a challenging task. Many factors play important roles and the accurate design of the system could become the key to obtain the desired outcomes. This integrated system should be capable of handling the large amounts of data obtained in a very short time from different vital sign sensors. Common sensors collect vital sign information such as heart rate, blood pressure, or electrocardiography (ECG). Also, the accurate design of this integrated mHealth system should address energy consumption issues in the network, the mobile computer, and the medical sensors [7]. Other import issues are the design and selection of the connectivity architecture and the communication technologies [7]. Meeting quality of service (QoS) requirements, in terms of the network parameters, ensures to have innovative, accurate, and cost-effective mHealth systems.  Figure 1 illustrates an example of mHealth architecture and infrastructure for emergency scenarios.

The aim of this paper is to systematically review the architectures, connective technologies, and energy optimization protocols implemented in the mHealth systems for emergency scenarios in outdoors in the last five years. There are several surveys and taxonomies about the deployed mHealth architectures for healthcare domain, and also, some studies are targeted for emergency scenarios. However, to the best of our knowledge, no systematic review has been conducted to overview the different types of mHealth architectures currently being implemented for emergency scenarios in outdoors. Moreover, our systematic review seeks for the efforts surrounding the mHealth systems to attend the patient in emergency situations in terms of architecture integration, communication, and energy optimization proposals. In that manner, this review collects valuable information about the following: (1) mHealth third-tier architecture for emergency scenarios in outdoors, (2) the traditional and emergent infrastructure connectivity technology between the three tiers, and (3) energy optimization protocols employed to increase the lifetime of the mHealth systems. The study concludes by discussing the future directions in the field.

## 2. Materials and Methods

This examination was conducted in September 2016–April 2017. The main goal was to find, study, and evaluate the recent efforts related to the infrastructure and architecture in favor of mHealth systems. This objective was fulfilled via the Internet by selecting the following databases: IEEE Xplore, Scopus, SpringerLink, ScienceDirect, and SAGE. The keywords used (employing Boolean phrases) in the searching were as follows: mobile health, m-health, mHealth, remote health, ubiquitous health, mobile healthcare, pervasive health, architecture, infrastructure, and emergency. Considering our interest to provide an analysis of novel productions, articles published after 2012 were considered. We included exclusively productions published in English and research from peer-reviewed journals. During the first stage, duplications were excluded and papers were analyzed by their title and abstract seeking for a match with our scope of interest. On a second stage, the remaining productions were subjected to a full-text review.

The initial query displayed 5801 results. After the first stage (title and abstract evaluation and elimination of duplicates), the number was reduced to 366. Once initiated the full-text review, we aimed our analysis in publications addressing the remote tracking of medical emergencies in outdoor scenarios, considering the importance previously described about traffic accidents or sudden-onset emergencies as a consequence of chronic degenerative assistance. The inclusion criteria were performed as follows: (i) articles including emergency events handling (e.g., wireless transmission of patient's vital signs in real time, alarm systems, emergency services automatic request, and patient's location via messages or calls); (ii) the study includes system and prototype developments, evaluation or validation of algorithms, protocols, models, and systems; (iii) the study was a peer-reviewed article published in journals; (iv) articles where approaches considered collecting data for at least one wireless sensor; (v) studies displaying evidence in the use of wireless technologies, that means, their infrastructure and architecture included two or more tiers; and (vi) articles were written in English language. In the course of full-text review exclusion criteria was determined by (i) articles derived from posters, glossaries, conferences, congresses, meeting, front covers, and book chapters; (ii) articles not written in English language; (iii) articles not supporting emergency situations; (iv) articles considering emergency management for hospital scenario; and (v) studies where the architecture for mHealth services included just organizational and conceptual models (lack of infrastructure in the deployment). All articles were evaluated looking for real evidence in the outcomes presented. After two stages of evaluation process, 25 articles were selected for the present analysis.

## 3. Results

In our review, we detected only 5 (20%) articles that are specific for outdoor environments. The other reviewed architectures 20 (80%) were targeted for indoors and outdoors. After the analysis of the selected articles, we extracted the main information of the recent architectures used for mHealth monitoring in emergency situations.  Table 1 summarizes the extracted information of the selected studies. The following sections present the results of (1) demographic data extracted from the articles, (2) the sensor, mobile, local, and remote units for the existing m-Health architectures in emergency scenarios, (3) the infrastructure connectivity between the three-tiers, and (4) the current energy optimization deployments.

### 3.1. Demographics

This section reports demographic data extracted from the 27 selected articles. The articles were from 20 different journals; the most common journal was International Journal of Distributed Sensor Networks 4 (16%).

**Figure 2:**
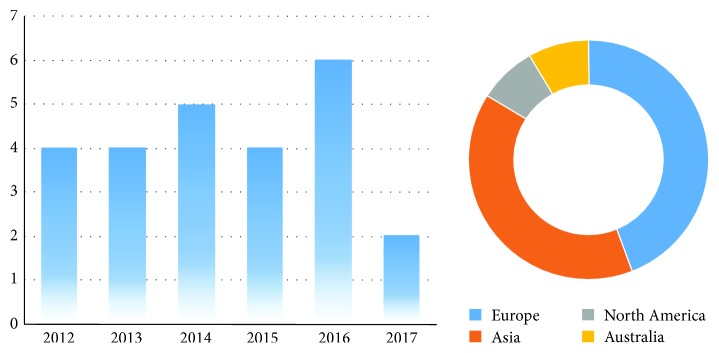
Frequency of the selected articles per year and per continent.

Figure 2 presents the frequency of the articles per year and per continent. As the data sample range was too short; it was not possible to consider possible trends. However, it was important to report that most of the selected articles were published in 2016. The graph also illustrates that most of the reviewed mHealth systems were from Europe 11 (44%), followed by Asia 10 (40%), North America 2 (8%), and Australia with 2 (8%) studies reported.

### 3.2. mHealth Architecture for Emergency Scenarios

The taxonomy of mHealth system architecture for emergency scenarios in outdoors is focused on the three tier structure: intra-WBSN communication for the first tier, inter-WBSN communication for the second tier, and beyond-WBSN communication for third tier.

#### 3.2.1. First Tier: Sensor Units

The first tier considers the implementation of a body sensor network (BSN). The sensors have a direct communication with the personal server (PS) implemented in the second tier. The main sensor units for mHealth systems in emergency scenarios in outdoors correspond to physiological and environmental sensors. The selected studies used a diverse type of physiological data to monitor the internal condition of the person; however, authors in [18] did not report the acquisition of physiological signals into the mHealth system architecture. The physiological signal most commonly found was electrocardiography (ECG). It was also common to find environmental sensors to monitor ambient conditions at the emergency location. The reported ambient conditions were temperature, light, noise, and humidity [10, 11, 20, 24]. In terms of sensor data sampling, authors in [8] reported AN error-free transmission of biomedical signals. An adaptive dithered signed-error constant modulus algorithm was implemented into the communication link to obtain a high correlation between original and transmitted data samples.

#### 3.2.2. Second Tier: Mobile Unit

The second tier considers the implementation of a PS (mobile unit). The PS has a direct communication with the database servers implemented in the third tier. Smartphones and tablets are the most common units used for mHealth systems. The main built-in technologies used by the smartphone users are the video and the global positioning system (GPS) technologies. The use of GPS technology is fundamental to locate the person with the emergency situation in outdoors. In [26], a smart and unified interface based on context-aware and adaptive interface is set for people with disabilities. RFID tags and Wi-Fi networks are used to track and determine the location of people with disabilities. In [28], the GPS is used for outdoor positioning of the patient; but for indoor positioning, a received signal strength indication (RSSI) is implemented into the WLAN. The framework switches between indoor and outdoor localization via one transmitted bit according to the mobility of the person.

The use of video technology is valuable for visual monitoring the scenario of the emergency in real time. In [11], video streams of multiple cameras were collected and multiplexed with other medical information. Ambient videos were collected and transmitted as the ambulance reaches the emergency area. While ambulance reaches emergency area, ambient videos were collected and transmitted. Inside the ambulance, paramedics can create videos about the condition of the patient; the specialized medical staff can later visualize the emergency situation. In [15], cameras made possible the interaction with the elders within ambient-assisted living environments. In [23], a wireless multimedia sensor network using a video recorder is applied in order to track patient's activity and medical condition. In [26], the authors use video technology with the intention to record disabled people in his/her working places. The analysis of these recorded videos facilitates the guidance and assistance of disabled people.

#### 3.2.3. Third Tier: Local and Remote Servers

The third tier considers the implementation of database servers in order to store, manage, and visualize the patient data. The main units used are local and remote servers. The most common deployments for mHealth systems for emergency scenarios in outdoors include the communication with the local server and the communication with the remote servers via the Internet. In [11, 15, 17, 24], the physiological and environmental data are only stored and visualized at the following local databases: monitoring center, hospital, caretaker site, and emergency center. In [16, 20, 22, 25, 27–31], the data is directly transmitted and managed by web services; in this way, the doctors, users, caretakers, and family receive alerts in case of emergency situations.

### 3.3. Infrastructure Connectivity

Infrastructure connectivity refers to the communication technology implemented between each tier of the mHealth system. The taxonomy proposed for this category includes the traditional connective technology and the emergent connective technology.

#### 3.3.1. Traditional Connective Technology

The traditional connective technology considers the available communication services to manage and transmit the information. Either wired or wireless sensors transmit the physiological information to a mobile device via Bluetooth or Zigbee, the mobile device sends the data through a cellular network or a local area network services, and finally, the data is managed, visualized, and stored into a local or a remote server.

#### 3.3.2. Emergent Connective Technologies

Nowadays, the information is being managed in different manners, not only via one-way traditional services. The emergent Internet connective technologies consider other types of manners to transmit, process, and manage the data, such as cloud services, Internet-of-things (IoT), distributed services, machine-to-machine (M2M), vehicular ad hoc network (VANET), and service-oriented architectures (SOA).

The most common implemented connective technology for mHealth systems in emergency scenarios in outdoors is the cloud services. In [13], a named data networking technology was implemented to deliver rich media contents from cloud, such as healthcare video adaptive streaming. In [20], cloud computing was used to provide the system with sensor data management and remote monitoring services. Cloud environment could provide a solution for reasoning component decisions and data mining. In [22], cloud services support a range of capabilities of storing, processing, and networking. Cloud data offers the possibility to scale up the system with the number of monitored patients, increasing size of data and interconnected components. In [28], an emerging cloud-supported cyber-physical system (CCPS) computing paradigm is implemented. The CCPS and smartphone integration solves many challenges regarding localization; with the use of the cyberspace, the complex processing task of localization could be done in real-time and efficient manner.

The combination of cloud services and IoT connective technologies is another alternative for mHealth systems. In [29–31], the synergy of IoT and cloud computing (CC) is implemented to obtain the sensor data of the IoT subsystem and use it by the cloud to analyze, process, and classify these physiological information in order to improve the accuracy, efficiency, and reliability of the framework. Additionally, the only use of IoT technology is reported in [16]. IoT enabled intelligence capabilities for real-time monitoring uninterruptedly, allowing emergencies to be detected immediately. The physical devices and patients became virtual entities and can be monitored from web services. In terms of distributed services such as connective technology, the implementation of a publish/subscribe internetworking (PSI) is reported in [17]. The network is based on the publish/subscribe paradigm: publishers providing information, subscribers consuming specific information, and an event notification service matching the advertised information. The PSI technology is introduced in assistive environments and used for an emergency event. The PSI publishes and controls patient data, a notification message advertises the data, and the healthcare unit examines and subscribes for the specific data of the emergency. In [26], a middleware distributed service architecture is implemented as a tool to abstract the hardware features and protocols from high-level layers due to the inexistence of devices and platforms standardization. In [14], the implementation of centralized and distributed services is reported. In this architecture, the connectivity is adaptable. In centralized mode, the WBSN only connects with the healthcare station. In distributed mode, the WBSN connects with the healthcare station and also sends the data to a medical display coordinator which visualize the information. Another important emergent technology is the implementation of M2M infrastructure. This technology offers a convergence between objects and intelligent services. An optimized scheme for movement coordination and data routing technique are introduced in [23]. The scheme includes a routing framework where several sensor devices can relay data to all receivers interested in that data. Finally, in [32], a hybrid Wi-Fi peer-to-peer (P2P) architecture is implemented to provide a high-quality health service that considers the mobility of the user and the optimization of the M2M data traffic via a dispersed cross-layer algorithm that optimizes the TCP/IP stack. In terms of VANET, the mobile network was used inside the ambulance to support mobile connectivity between the hospital and the ambulance in case of critical cases [10]. Finally, a SOA connective technology is applied in [27] to deal with communication issues and extend interoperability and usability including microblogging services (Twitter) whereby patient's community could be informed of patient's medical status.

### 3.4. Energy Consumption and mHealth System Optimizations

It is important to take into account the lifetime of the mHealth systems especially when these systems are intended for emergency scenarios, in which the data transmission is demanding, in real time and critical. Moreover, for outdoor environments, the mobility of the platforms prevents the components of the system (sensors, smartphones) to be energized at any time, so the lifetime of the system must be sustainable.

#### 3.4.1. Implemented Energy Consumption Protocols

Although energy optimization procedures were proposed for increasing the lifetime of the mHealth systems in emergency scenarios in outdoors, few studies implemented an energy consumption protocol. In [12], a lifetime optimization algorithm was implemented to maximize the minimum lifetime of each sensor in the WBSN. The disadvantage reported is that in case of an emergency the WBSN information is transmitted regardless of the energy consumption. In [13], an energy consumption awareness is evaluated in for LTE and Wi-Fi technologies. In addition, the implementation of NDN technology saves energy and other resources in the network. In [14], an energy-aware peering routing protocol (EPR) is used to reduce the traffic load and energy consumption choosing the nearest node available. The drawback is the implementation of the EPR as an indoor scenario. In [29], a fuzzy-based data fusion technique is implemented in order to save bandwidth. The method reduces the transmission and processing of the sensed data to remove redundant information from the sensors, thus increasing the network lifetime. In [32], a dispersed cross-layer optimization algorithm is used in order to improve the efficiency and reliability of the network. One drawback is that each wireless device needs to interoperate and the algorithm should differ depending on the network environment, but this could be solved with a dynamic configuration for the MAC layer.

#### 3.4.2. Not Implemented Energy Consumption Protocols

The other studies only suggested that some actions could be taken to improve battery lifetime. In terms of minor data transmission, [9, 15, 16, 25, 31] reported that only the relevant information such as alarms could be transmitted. It could reduce the amount of data transmission between the sensors and the mobile devices, resulting in battery savings. In terms of controlling the idle state of the transmitter, [10, 19] reported that a protocol could turn-off the sensor node while inactive and/or maintain the transmitter idle when no data is available. It could save the battery consumption. In terms of efficient communication protocol, [31] mentioned the opportunity to use a simplified protocol stack into the Bluetooth interface. In terms of energy-aware monitoring, [22] considers the network resources, memory availability, processing computation, and battery drainage. Finally, [23] proposes an energy-aware routing technique for data transmission allowing to choose a path with maximal residual energy and at a time the path with minimal energy consumption.

## 4. Discussion

Architecture and infrastructure for mHealth services vary depending on the different ways to deploy a wireless sensor network for emergency scenarios in outdoors. After analyzing 25 articles, our review process pointed out that the majority of the reviewed mHealth systems were intended for patients suffering from either cardiac, epilepsy, dementia, or paralysis disease. Also, the elderly was the predominant population, and the athletes and disabled people represented the secondary population.

In the majority of the revised articles, 80%, the mHealth systems were intended for indoors and outdoors; however, the deployment and evaluation of these systems were only for indoor scenarios. This finding highlights the need to show real evidence of mHealth systems supporting emergencies in outdoors scenarios. The need of mhealth systems deployed for outdoors along with two key factors makes evident our decision to highlight how wireless body sensor networks (WBSN) are handling emergency events. The key factors are the need for emergency care for patients within the golden hour and the previously stated increment in the rate of traffic accidents [23]. Our review made also possible the identification of the following important facts related to the use of WBSN for emergency care in outdoors scenarios. In terms of sensor units, there is a large amount of data generated due to the send-data frequency under emergency events and the diversity of the acquired signals: physiological and environmental. It is reported that under emergency situations; the personal health information is updated, at least, every 10 seconds [5]. To handle this situation, sensors should be categorized by priority according to the critical condition suffered by patients (heart attack, stroke, respiratory arrest, etc.). Accurate scheduling techniques should be part of the routing protocols at the system's medium access control layer to avoid data loss due to several sensors trying to access the communication channel at the same time. Possible techniques identified are smart energy-aware sensors that are only activated at specific time slots; intelligent algorithms that take into account conditions like abnormal data, buffer space remaining, energy constraints, and physiological parameter priorities [33]; and fuzzy logic algorithms that decide the best time to start a data transmission [34]. In terms of video technology, physicians are more comfortable if they can visualize patient's condition. The use of video streams is a strong tool to help physicians for an accurate diagnosis. Factors such as patient's coloring, muscle stiffness, state of shock evidence, and rapid breathing are only some of the significant conditions that could be appreciated by physicians through video technologies. In terms of connective infrastructure, the traditional technologies are being overcome by the following emergent technologies: cloud services, distributed services, and IoT. As the accessibility to the information is an important feature for mHealth systems, these emergent technologies provide the architectures with resourceful capabilities for different kind of services. Another important consideration is that the WBSN-based designs have to take into account factors like mobility, infrastructure at urban and rural areas, and crowded environments with the coexistence of different communication technologies. One of the most important features of mHealth systems is the energy consumption. Important energy optimizations to be considered are (1) the incorporation of signal-processing techniques to reduce data volume; (2) the incorporation of vital sign correlation to improve the communication network performance; and (3) the adoption of sampling strategies to reduce the data volume. Signal processing reduces the data volume using compression techniques. The reduction in energy consumption occurs if the time to transmit compressed data is shorter than the time to transmit uncompressed data. Relevant signal-processing techniques within WBSN are QR peak detection, QRS complex, interpolation algorithms, and compressive sensing. The reduction in energy consumption is also possible by correlating vital sign data and by incorporation polling intervals at the sensors, only after the identification of an abnormal condition during the correlation process. Reductions in energy consumption are also possible by adopting sampling strategies that avoid a constant sampling rate [35]. The correct selection of a sampling strategy can reduce data volume and the energy consumption at the time to transmit data over a WBSN. Other sampling strategies that must be considered to improve energy efficiency are (1) adaptive sampling that modifies in real time the data collection after analyzing the correlation between the sensed data and the energy remaining at the power supply; (2) hierarchical sampling with multiple complex and simple sensors enabling the possibility to trade precision and power consumption; and (3) model-driven active sampling that builds an abstraction of the sensed parameter using a forecasting model. This model predicts the next data to be acquired by the sensor.

A final WBSN-based design and implementation has to consider energy efficiency. The whole WBSN-based system can fail if one or more nodes run out of battery. The WBSN-based design has to take into account consumption-aware policies and adaptive behaviors regarding network and patient conditions [33, 36].

## 5. Conclusion

Our systematic review presented a comprehensive overview of the integrated mHealth architectures for outdoor emergencies. Our systematic review also highlighted the emergent connectivity technologies and the energy optimization protocols implemented in mHealth systems for outdoor emergencies.

Despite the growing presence of remote health monitoring in home, hospital, and emergency scenarios, the development based on wireless body sensor networks (WBSN) is still at a redefinition stage especially for outdoor emergencies.

Our review also highlighted the need for more integrated mHealth solutions specifically for outdoor scenarios. Only one of our surveyed studies presented an integrated solution based on the need of mHealth systems capable of switching among requirements depending on an outdoor or indoor context. Also, the integration of emergent connective technologies into mHealth architectures are redefining the way information is being managed. Emergent technologies such as cloud services and Internet-of-things (IoT) represent new paradigms for the communication among specific layers found in mHealth systems. The sensor nodes demanded by these technologies must satisfy several requirements: (1) high degree of integration with a small size, (2) local processing of multiple signals, (3) reduction in power consumption of the microprocessor, (4) reduction in wireless data transmission, (5) data intensive collection, and (6) reduced duration of the processor's active mode. Finally, our review highlighted the need for implementing and evaluating energy consumption protocols.

## Figures and Tables

**Table 1:** Summary of the recent architectures for m-Health in emergency situations.

Ref.	Emergency context	Target loc.	1st tier		2nd tier		3rd tier		Energy opt.	Infras. connec.	Type of dev.
			Sensors	Com. tech.	Mobile unit	Com. tech.	Local serv.	Rem. serv.			
[8]	Direct transmission of ECG through mobile network where the nearest medical center can be contacted, and by GPS, the patient can be found and saved.	Outdoors	ECG EEG	Bluetooth or Zigbee	Smartphone with GPS	GSM or EDGE/Bluetooth or Wi-Fi	Base station	Networked PC → Internet → healthcare station	—	Traditional	Algorithm evaluation

[9]	A personal emergency response system with an automatic detection on falls to send an alarm to the caregivers.	Indoors/outdoors	ACC	Bluetooth	Smartphone with internal ACC, GPS, and GUI	—	—	—	Can be reduce the transmission 1st-2nd tiers. 1st detects falls instead of 2nd.	Traditional	System reliability

[10]	Abnormal medical parameters are sent immediately to medical facilities by reserving priority slots to report them in real time.	Indoors/outdoors	Phys. Environ. PAN coord	Bluetooth or Zigbee	Smarthpone or tablet with GPS and GUI	GSM, 3G, or 4G → Ambulance → VANET	Hospital server	LAN, WLAN → Remote database or Doctor's PDA	Power management mechanism. Efficient MAC protocol to control sensor's activity	VANET	Model proposal and algorithm evaluation

[11]	Multiple videos and medical data are multiplexed and transmitted in real time to the hospital. Medical staff can coordinate, visualize, and prearrange treatment.	Indoors/outdoors	Ambient videos	—	Ambulance with cameras, GPS, USG, and vital signs	LTE	Hospital center	—	—	Traditional	Algorithm evaluation

[12]	Emergency medical information is transmitted directly through a cellular network without multihop communications. It is critical to allocate a dedicated channel to the base station.	Indoors/outdoors	EEG ACC ECG Temp. BP EMG Glucose	Bluetooth	Smartphone	Cellular network or Wi-Fi	Base station	AP → Internet → Doctor's PDA	Determine dynamically the number of channels to efficiently utilize the resources of the network	Traditional	Energy scheme evaluation

[13]	Patient's vital signs are transmitted simultaneously during a disaster to the medical center for fast analysis and diagnosis.	Indoors/outdoors	HR RR BP SpO2	Zigbee	Smartphone	LTE or Wi-Fi	Base station	AP → Internet → Emergency center	NDN reduce upstream traffic to efficiently utilize the resources of the network	Cloud services	Protocol network evaluation

[14]	In the hospital emergency room, the BAN patient data is displayed in real time to the medical coordinator. Patient may be transferred to further treatment.	Indoors/outdoors	EEG Positioning ACC SpO2 EMG Glucose BP Hearing	MAC IEEE 802.15.4	BAN coordinator	Internet	Database server	Emergency ambulance device provider system	Energy-aware peering routing protocol	Centralized and distributed services	Routing protocol implement.

[15]	Wearable light device (WLD) detects an abnormal event. Mobile phone reports an alarm signal to caretaker without user interaction. The caretaker could request further measurements.	Indoors/outdoors	ECG SpO2 PPG Temp. ACC WLD coord. Videoconf. GPS	Bluetooth	Smartphone	—	Caretaker site	—	Only transmits alarms to reduce data transmission	Traditional	System dev.

[16]	In a web portal to real-time monitoring of health status of elderly, their families or clinicians can visualize a person's location and status of alarm of abnormal physiological parameters.	Indoors/outdoors	ECG Temp. ACC Glucose EMG SpO2 Arduino coord	Bluetooth	Smartphone with GPS and RFID tag	Wi-Fi or LTE	—	Web services → Healthcare management system	Fuzzy logic to decide events and minimize communication resources	Internet-of-things	System implement.

[17]	Heart rate signals are available through PSI network. Alarm is generated and a nearby hospital is notified. The system provides access to EHR.	Indoors/outdoors	ECG Arduino coord	Zigbee or Bluetooth or Wi-Fi	Smartphone	PSI	Storage and visual. device	—	—	Distributed services	Conceptual and prototype implement.

[18]	If the m-Carer system detects a critical situation, it sends a message to the location server and to the emergency service to inform the location of the patient.	Outdoors	—	—	Smartphone with GPS	Wi-Fi or 3G	Emergency service	Location and preference servers → Internet → Doctors, relatives	—	Traditional	System dev.

[19]	If an abnormal condition is detected such as critical cardiac event, patients are referred to the emergency department.	Indoors/outdoors	ECG	Bluetooth	Smartphone	3G or Wi-Fi	Clinical decision support	Networked PC → Internet → Doctor's PDA	BATCH transmission to improve battery duration versus greedy transmission node	Traditional	System dev.

[20]	CARA system reasoning is capable to predict possibly risky situations by correlating different sensor readings over time and to notify the emergency.	Indoors/outdoors	ECG RR HR BP ACC Environ.	Bluetooth	Smartphone	—	—	Web services → Internet → Emergency services, caregivers	—	Cloud services	System implement.

[21]	After detection of a fall or cardiac anomaly, alert enabler system automatically contact patient's family or emergency services.	Outdoors	Biomedical	Bluetooth	Smartphone with GPS	3G or Wi-Fi	Local database	Remote database	—	Traditional	App dev.

[22]	The system monitors continuously the patient's health condition, and if an emergency situation is detected, a request for ambulance is sent.	Outdoors	ECG EEG	Bluetooth	Tablet	3G, 4G or Wi-Fi	—	Web services → Internet → Doctor's device	Energy and resource-aware monitoring	Cloud services	Model evaluation and implement.

[23]	A system allowing real-time analysis of sensors' data, providing guidance and feedback to the user. This approach generates warnings based on the user's state, level of activity, and environmental conditions related to patients.	Indoors/outdoors	BP HR ECG EKG Temp. Video Audio GPS	M2M Area network	Mobile devices, M2M gateway	M2M Communication technology (Internet)	Database server	Doctor's PDA, insurance companies.	Optimization scheme for movement coordination technique and data routing within the monitored area.	M2M	Movement tracking algorithm evaluation

[24]	Sports environment monitoring system. An alarm is sent if any hazardous condition appears or any of the defined user upper or lower thresholds are surpassed.	Outdoors	HR RR ACC Temp Environ.	Bluetooth	Smartwatch or smartphone	Internet (technology not specified)	Emergency services	—	—	Distributed services	Wireless sensor network app develop.

[25]	A system is developed through a program running on a smartphone integrated with Bluetooth. The smartphone is able to receive data from sensors in order to detect fall and issue alarm.	Indoors/outdoors	ACC Gyroscope GPS	Bluetooth	Smartphone	3G/GSM		Family and Healthcare providers	Efficient learning algorithm with the highest accuracy running on a cell phone	Traditional	System develop.

[26]	A context-aware smart solution able to track and determine disabled user's location. If the user is having a critical health issue, a message will be sent to a caregiver.	Indoors/outdoors	RFID tags (for user's movements) GCS BS Video Hearing	RFID/Wi-Fi	APs Smartphone	Wi-Fi	Database server	Caregiver's PDA/smartphone	Use of RFID technology	Traditional	System develop.

[27]	A pervasive health system enabling self-management of chronic patients during their everyday activities.	Indoors/outdoors	HR SpO2 BP Weight	Bluetooth	Smartphone (mobile base unit)	3G/4G HTTP	—	Medical center Web-based microblogging services (Twitter)	—	SOA	System develop.

[28]	Abnormal detection while a heart attack, the smartphone sends the data along with the patient's location to the remote cloud. A decision support system notifies entrusted emergency centers.	Indoors/outdoors	EEG	Bluetooth	Smartphone with microphone and GPS	UWB, RSSI on WLAN, LBS, Wi-Fi, 4G, and/or RFID	—	Web services → Emergency services, doctors	—	Cloud services	System evaluation

[29]	A patient suffering from CHF is continuously monitored. The cloud services generate alarms to ER services when abnormalities are detected.	Indoors/outdoors	Glucometer CO2 level SpO2 ECG	Bluetooth	Tablet or smartphone with GPS	Wi-Fi, LTE	—	Web services → MC, doctors, ambulance	A fuzzy-based data fusion technique to remove redundant data	IoT and cloud services	System evaluation

[30]	In a smart fall detection system, the patient monitored for mobility and fall events are reported as alarms.	Indoors/outdoors	ACC	Bluetooth	Smartphone	Cellular network	—	Web services	—	IoT and cloud services	Algorithm evaluation

[31]	Emergency is detected and notified by appropriate ringtone or vibration patterns in the smartphone. Also, alerts are sent to the rescuers.	Indoors/outdoors	PPG	Bluetooth	Smartphone	Wi-Fi, 2G, 3G, 4G	—	Web services → Doctors, users	Efficient Bluetooth low energy protocol stack	IoT and cloud services	Prototype implement.

[32]	Different levels of attention but the patient is monitored continuously, and in mobility, it can respond to emergency situations.	Indoors/outdoors	Blood pressure ECG ACC Temperature	Wi-Fi with P2P architecture	Smartphone	Wi-Fi with P2P architecture	Base station	Hospital, doctor's PC	A dispersed cross-layer optimization algorithm to optimize resources in the network	M2M	Model evaluation and implement.

*Note*. Ref. = reference; Loc. = location; Com. = communication; Tech. = technology; Serv. = Server; Opt. = optimization; Infras. = infrastructure; Connec. = connectivity; Dev. = development; ECG = electrocardiography; GPS = global positioning system; EEG = electroencephalography; PC = personal computer; ACC = accelerometer; GUI = graphic user interface; PR = pulse rate; Temp = temperature; SpO2 = oxygen saturation; BP = blood pressure; Phys. = Physiological; Environ. = environmental; NDN = named data networking; PAN = personal area network; coord = coordinator; PDA = personal digital assistant; VANET = vehicular ad hoc network; USG = ultrasonography; EMG = electromyography; AP = access point; BAN = body area network; HR = heart rate; RR = respiratory rate; implement. = implementation; PPG = photoplethysmography; Videoconf = videoconference; perform. = performance; EHR = electronic health record; M2M = machine to machine; GCS = gesture control sensor; BS = behavior sensors; UWB = ultrawideband; RSSI = received signal strength indication; LBS = location-based services; CHF = congestive heart failure; MC = monitoring center; P2P = peer-to-peer; SOA = service-oriented architecture.
